# Multi-omic Analyses Shed Light on The Genetic Control of High-altitude Adaptation in Sheep

**DOI:** 10.1093/gpbjnl/qzae030

**Published:** 2024-04-02

**Authors:** Chao Li, Bingchun Chen, Suo Langda, Peng Pu, Xiaojia Zhu, Shiwei Zhou, Peter Kalds, Ke Zhang, Meenu Bhati, Alexander Leonard, Shuhong Huang, Ran Li, Awang Cuoji, Xiran Wang, Haolin Zhu, Yujiang Wu, Renqin Cuomu, Ba Gui, Ming Li, Yutao Wang, Yan Li, Wenwen Fang, Ting Jia, Tianchun Pu, Xiangyu Pan, Yudong Cai, Chong He, Liming Wang, Yu Jiang, Jian-Lin Han, Yulin Chen, Ping Zhou, Hubert Pausch, Xiaolong Wang

**Affiliations:** International Joint Agriculture Research Center for Animal Bio-Breeding, Ministry of Agriculture and Rural Affairs/Key Laboratory of Animal Genetics, Breeding and Reproduction of Shaanxi Province, College of Animal Science and Technology, Northwest A&F University, Yangling 712100, China; Animal Genomics, ETH Zürich, Zürich 8092, Switzerland; International Joint Agriculture Research Center for Animal Bio-Breeding, Ministry of Agriculture and Rural Affairs/Key Laboratory of Animal Genetics, Breeding and Reproduction of Shaanxi Province, College of Animal Science and Technology, Northwest A&F University, Yangling 712100, China; Institute of Animal Science, Tibet Academy of Agricultural and Animal Husbandry Sciences, Lhasa 850009, China; School of Biological and Pharmaceutical Engineering, Lanzhou Jiaotong University, Lanzhou 730070, China; College of Life Sciences, Shaanxi Normal University, Xi’an 710119, China; International Joint Agriculture Research Center for Animal Bio-Breeding, Ministry of Agriculture and Rural Affairs/Key Laboratory of Animal Genetics, Breeding and Reproduction of Shaanxi Province, College of Animal Science and Technology, Northwest A&F University, Yangling 712100, China; International Joint Agriculture Research Center for Animal Bio-Breeding, Ministry of Agriculture and Rural Affairs/Key Laboratory of Animal Genetics, Breeding and Reproduction of Shaanxi Province, College of Animal Science and Technology, Northwest A&F University, Yangling 712100, China; International Joint Agriculture Research Center for Animal Bio-Breeding, Ministry of Agriculture and Rural Affairs/Key Laboratory of Animal Genetics, Breeding and Reproduction of Shaanxi Province, College of Animal Science and Technology, Northwest A&F University, Yangling 712100, China; Animal Genomics, ETH Zürich, Zürich 8092, Switzerland; Animal Genomics, ETH Zürich, Zürich 8092, Switzerland; International Joint Agriculture Research Center for Animal Bio-Breeding, Ministry of Agriculture and Rural Affairs/Key Laboratory of Animal Genetics, Breeding and Reproduction of Shaanxi Province, College of Animal Science and Technology, Northwest A&F University, Yangling 712100, China; International Joint Agriculture Research Center for Animal Bio-Breeding, Ministry of Agriculture and Rural Affairs/Key Laboratory of Animal Genetics, Breeding and Reproduction of Shaanxi Province, College of Animal Science and Technology, Northwest A&F University, Yangling 712100, China; Institute of Animal Science, Tibet Academy of Agricultural and Animal Husbandry Sciences, Lhasa 850009, China; College of Life Sciences, Shaanxi Normal University, Xi’an 710119, China; College of Life Sciences, Shaanxi Normal University, Xi’an 710119, China; Institute of Animal Science, Tibet Academy of Agricultural and Animal Husbandry Sciences, Lhasa 850009, China; Institute of Animal Science, Tibet Academy of Agricultural and Animal Husbandry Sciences, Lhasa 850009, China; Institute of Animal Science, Tibet Academy of Agricultural and Animal Husbandry Sciences, Lhasa 850009, China; Zoology and Evolutionary Biology, Department of Biology, University of Konstanz, 78457 Konstanz, Germany; College of Life and Geographic Sciences, Kashi University, Kashi 844000, China; International Joint Agriculture Research Center for Animal Bio-Breeding, Ministry of Agriculture and Rural Affairs/Key Laboratory of Animal Genetics, Breeding and Reproduction of Shaanxi Province, College of Animal Science and Technology, Northwest A&F University, Yangling 712100, China; International Joint Agriculture Research Center for Animal Bio-Breeding, Ministry of Agriculture and Rural Affairs/Key Laboratory of Animal Genetics, Breeding and Reproduction of Shaanxi Province, College of Animal Science and Technology, Northwest A&F University, Yangling 712100, China; Beijing Key Laboratory of Captive Wildlife Technologies, Beijing Zoo, Beijing 100044, China; Beijing Key Laboratory of Captive Wildlife Technologies, Beijing Zoo, Beijing 100044, China; Department of Medical Research, Guangdong Provincial People’s Hospital, Guangdong Academy of Medical Sciences, Guangzhou 510080, China; International Joint Agriculture Research Center for Animal Bio-Breeding, Ministry of Agriculture and Rural Affairs/Key Laboratory of Animal Genetics, Breeding and Reproduction of Shaanxi Province, College of Animal Science and Technology, Northwest A&F University, Yangling 712100, China; Key Laboratory of Agricultural Internet of Things, Ministry of Agriculture and Rural Affairs/Shaanxi Key Laboratory of Agricultural Information Perception and Intelligent Service, College of Information Engineering, Northwest A&F University, Yangling 712100, China; Institute of Animal Husbandry and Veterinary Medicine, Xinjiang Academy of Agricultural and Reclamation Sciences, Shihezi 832000, China; State Key Laboratory of Sheep Genetic Improvement and Healthy Production, Xinjiang Academy of Agricultural and Reclamation Sciences, Shihezi 832000, China; International Joint Agriculture Research Center for Animal Bio-Breeding, Ministry of Agriculture and Rural Affairs/Key Laboratory of Animal Genetics, Breeding and Reproduction of Shaanxi Province, College of Animal Science and Technology, Northwest A&F University, Yangling 712100, China; CAAS-ILRI Joint Laboratory on Livestock and Forage Genetic Resources, Institute of Animal Science, Chinese Academy of Agricultural Sciences, Beijing 100193, China; Livestock Genetics Program, International Livestock Research Institute, Nairobi 00100, Kenya; International Joint Agriculture Research Center for Animal Bio-Breeding, Ministry of Agriculture and Rural Affairs/Key Laboratory of Animal Genetics, Breeding and Reproduction of Shaanxi Province, College of Animal Science and Technology, Northwest A&F University, Yangling 712100, China; Institute of Animal Husbandry and Veterinary Medicine, Xinjiang Academy of Agricultural and Reclamation Sciences, Shihezi 832000, China; State Key Laboratory of Sheep Genetic Improvement and Healthy Production, Xinjiang Academy of Agricultural and Reclamation Sciences, Shihezi 832000, China; Animal Genomics, ETH Zürich, Zürich 8092, Switzerland; International Joint Agriculture Research Center for Animal Bio-Breeding, Ministry of Agriculture and Rural Affairs/Key Laboratory of Animal Genetics, Breeding and Reproduction of Shaanxi Province, College of Animal Science and Technology, Northwest A&F University, Yangling 712100, China

**Keywords:** Environmental adaptation, High altitude, Hypoxia, Selection signature, Sheep

## Abstract

Sheep were domesticated in the Fertile Crescent and then spread globally, where they have been encountering various environmental conditions. The Tibetan sheep has adapted to high altitudes on the Qinghai-Tibet Plateau over the past 3000 years. To explore genomic variants associated with high-altitude adaptation in Tibetan sheep, we analyzed Illumina short-reads of 994 whole genomes representing ∼ 60 sheep breeds/populations at varied altitudes, PacBio High fidelity (HiFi) reads of 13 breeds, and 96 transcriptomes from 12 sheep organs. Association testing between the inhabited altitudes and 34,298,967 variants was conducted to investigate the genetic architecture of altitude adaptation. Highly accurate HiFi reads were used to complement the current ovine reference assembly at the most significantly associated β-globin locus and to validate the presence of two haplotypes A and B among 13 sheep breeds. The haplotype A carried two homologous gene clusters: (1) *HBE1*, *HBE2*, *HBB*-like, and *HBBC*, and (2) *HBE1*-like, *HBE2*-like, *HBB*-like, and *HBB*; while the haplotype B lacked the first cluster. The high-altitude sheep showed highly frequent or nearly fixed haplotype A, while the low-altitude sheep dominated by haplotype B. We further demonstrated that sheep with haplotype A had an increased hemoglobin–O_2_ affinity compared with those carrying haplotype B. Another highly associated genomic region contained the *EGLN1* gene which showed varied expression between high-altitude and low-altitude sheep. Our results provide evidence that the rapid adaptive evolution of advantageous alleles play an important role in facilitating the environmental adaptation of Tibetan sheep.

## Introduction

An agropastoral lifeway that emerged in the late Holocene enabled the permanent human occupation of the Qinghai-Tibet Plateau (QTP) [1,2]. Evidence from ancient DNA and archaeological remains indicated that Tibetan sheep populated on the QTP from its northeastern part ∼ 3100 years ago [3–5]. Transhumant pastoralism is common on the QTP, where livestock graze and migrate closely with humans, providing a wide variety of animal-based products to indigenous Tibetans [6,7]. Sheep are the most prevalent livestock on the QTP (> 20 million) [8], and Tibetan sheep are well adapted to the mountainous and hypoxic environments on the QTP [9].

Human and animal populations evolved convergently to cope with the harsh conditions in high-altitude environments [10]. Adaptation to high altitudes is a polygenic trait. Whole-genome scans have identified causative sequence variants that confer adaptability through various mechanisms in several species, including humans [11–14], monkeys [15–16], dogs [17–19], pigs [20], cattle [21], chickens [22,23], antelopes [24], and goats [25–28]. Variants in *EPAS1*, *EGLN1*, and *MKL1* are associated with responses to low oxygen conditions at high altitudes [11,13,29]. In contrast, variants in *GNPAT*, *CDT1*, and *MMP3* enhance tanning ability, thus protecting skin from increased ultraviolet (UV) radiation at high altitudes [15,30,31]. In Tibetan sheep, previous studies have revealed signatures of high-altitude adaptation that encompass functional genes associated with hypoxia and UV signaling pathways [*e.g.*, *HBB* and *MITF*, as well as the hypoxia-inducible factor 1 (HIF-1) pathway] [4,32,33]. However, the causative variants and their genetic mechanisms that confer high-altitude adaptability in sheep are still elusive.

Here, we utilized whole-genome sequence data from 924 domestic and 70 wild sheep inhabiting different ecosystems as well as 96 transcriptomes from 12 organs of sheep raised at different altitudes to explore the signatures of high-altitude adaptation. We investigated top-ranking selected signals related to adaptation to high altitudes, explored transcriptional regulatory changes, inferred sources of adaptation, and identified genes or likely causative variants associated with high-altitude adaptation in Tibetan sheep.

## Results

### Genome sequencing and population genetic structuring

**Figure 1:**
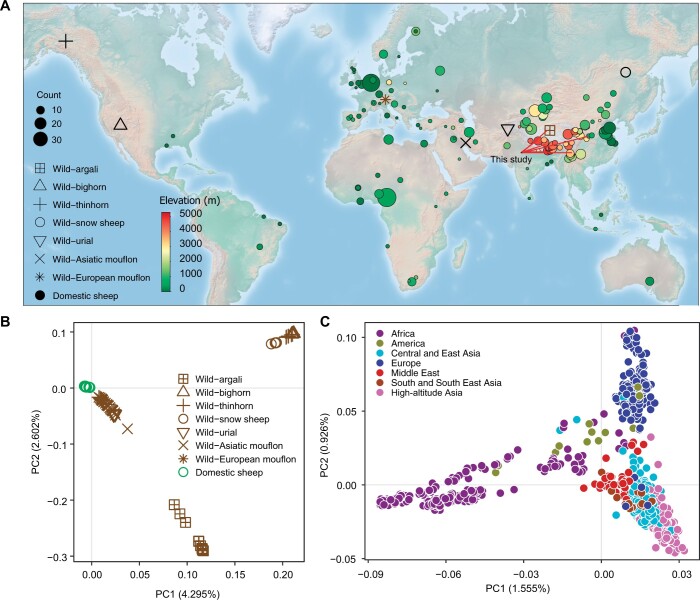
Geographical distribution and genetic diversity of 924 domestic and 70 wild sheep used in this study **A**. Geographical distribution of all sheep used in this study. The size of the solid circle represents the number of domestic sheep, while the shape indicates the distribution of wild individuals in a geographical region. Red lines indicate the locations of newly sequenced samples. **B**. and **C**. PCA of all samples (B) and domestic sheep only (C). PCA, principal component analysis; PC, principal component.

The whole genomes of 76 domestic sheep and one wild sheep (argali, *Ovis ammon*) were sequenced with short paired-end libraries to an average coverage of 21.37× (ranging from 15.23× to 38.49×) (Table S1). We also downloaded complementary data from public repositories and compiled a collection of 924 domestic and 70 wild sheep inhabiting different ecosystems at varying altitudes (**Figure 1**A; Table S2) [4,34–38]. The wild relative species included bighorn (*Ovis canadensis*, *n* = 6), thinhorn (*Ovis dalli*, *n* = 6), argali (*O. ammon*, *n* = 11), mouflon (*Ovis gmelini*, *n* = 33), urial (*Ovis vignei*, *n* = 6), and snow sheep (*Ovis nivicola*, *n* = 8). The sequence data were aligned to the current domestic sheep reference genome (ARS-UI_Ramb_v2.0, GCA_016772045.1) [39], and variant calling with the Genome Analysis Toolkit (GATK; v4.1.7.0) [40] yielded 141,008,381 single nucleotide polymorphisms (SNPs).

A principal component analysis (PCA) of the sequence variant genotypes grouped the sheep into three major clusters: (1) the bighorn, thinhorn, and snow sheep; (2) argali; and (3) domestic sheep, Asiatic mouflon, European mouflon, and urial (Figure 1B). Another PCA that included only domestic sheep separated most individuals by their geographic origins (Figure 1C). The observed dispersion pattern was supported by the phylogeographic pattern (Figure S1; Table S3) and agreed well with previous studies [32,35]. Asian sheep formed different sub-clusters based on the geographical locations and/or altitudes where they inhabited (Figure S2A). Additionally, the 145 Tibetan sheep representing 11 populations were divided into 3 sub-clusters following their isolated geographical distributions on the QTP (Figure S2B).

### Genome-wide selection for high-altitude adaptation

**Figure 2:**
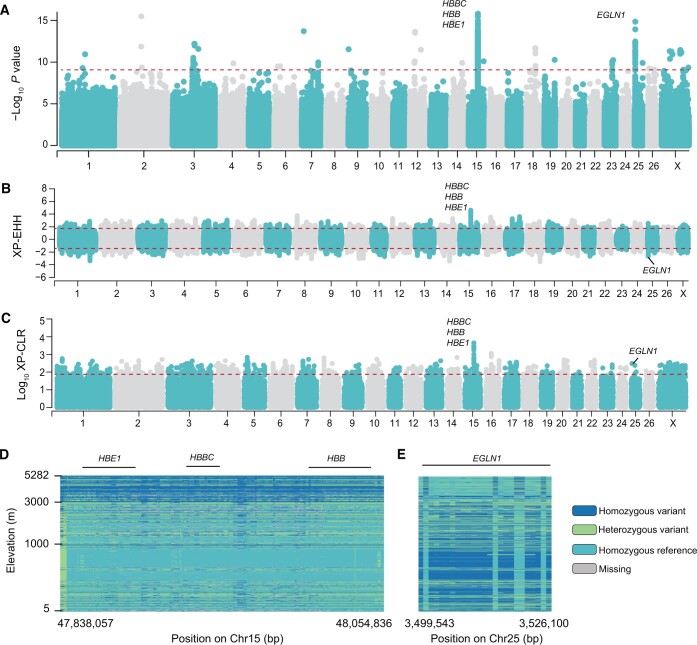
Association analysis between 34,298,967 variants and habitat altitudes in 450 sheep **A**. Manhattan plot representing the association of SNPs and INDELs (< 50 bp) with habitat altitudes in sheep (*n* = 450). The horizontal red dotted line indicates the Bonferroni-corrected significance threshold (−log_10_*P* value = 8.84). **B**. and **C**. Manhattan plots of genome-wide XP-EHH (B) and XP-CLR (C) estimates. The XP-EHH and XP-CLR estimates were calculated in 50-kb windows sliding in 10-kb steps along the genomes. The threshold values corresponding to the top 1% of the XP-EHH values (XP-EHH < −1.74 or XP-EHH > 1.99) and the XP-CLR values (−log_10_ XP-CLR > 1.943) are shown as the horizontal red dotted lines. **D**. and **E**. Comparison of selected genomic regions on Chr15 (D) and Chr25 (E) identified in selection scans of 924 domestic sheep. Chr, chromosome; SNP, single nucleotide polymorphism; INDEL, insertion and deletion; *HBE1*, hemoglobin subunit ε 1; *HBB*, hemoglobin subunit β-A; *HBBC*, hemoglobin subunit β-C; *EGLN1*, Egl-9 family hypoxia-inducible factor 1.

Animal populations that are adapted to high altitudes have evolved unique physiological responses driven by the genetic signatures of selection in their genomes. To characterize such signatures and linked functional variants in Tibetan sheep genomes, we used the habitat altitudes of 450 sheep as quantitative phenotypes in a mixed model-based association study based on 34,298,967 biallelic variants (**Figure 2**A). Of these variants, 896 [SNPs and insertions and deletions (INDELs) < 50 bp] reached genome-wide significance at a Bonferroni-corrected threshold of *P* < 1.46 × 10^−9^ (Figure 2A; Table S4), while most of them (*n* = 813/896, 90.74%) clustered in two genomic regions, one on chromosome 15 (*n* = 788, including four non-synonymous mutations) and another on chromosome 25 (*n* = 25). This observation was corroborated by the cross-population extended haplotype homozygosity (XP-EHH) analysis (Figure 2B) and the multilocus test of allele frequency differentiation (XP-CLR) (Figure 2C).

The region on chromosome 15 encompassed the β-globin locus (*P* = 1.59 × 10^−16^) (Figure 2D). In this region, two families of genes encoding two embryonic hemoglobins (Hbs) [Hb subunit ε 1 (*HBE1*) and Hb subunit ε 2 (*HBE2*), alongside their homologous HBE1-like and *HBE2*-like] and three β-Hbs [Hb subunit β-A (*HBB*) alongside its homologous *HBB*-like, *HBB1*-like, and *HBB2*-like; Hb subunit β-C (*HBBC*); and Hb fetal subunit β-F (*HBBF*)] were annotated in the current sheep reference genome (ARS-UI_Ramb_v2.0), which were highly similar to haplotype A at this locus [41]. This region was previously found to be associated with high-altitude adaptation in sheep, based on an earlier version of the sheep reference genome Oar_v4.0 (GCA_000298735.2) assembled from a Texel sheep [4], in which the β-globin locus belonged to the haplotype B that missed the four genes of *HBE1*, *HBE2*, *HBB*-like, and *HBBC* [41]. The region on chromosome 25 contained the Egl-9 family hypoxia-inducible factor 1 (*EGLN1*) gene (*P* = 1.41 × 10^−15^) (Figure 2E). Notably, *EGLN1* is involved in high-altitude adaptation in humans [11,42]. The haplotype diversity in these two regions separated the 924 domestic sheep according to their altitudes (Figure 2D and E, Figure S3).

### Global gene expression in 12 organs of high-altitude and low-altitude sheep

**Figure 3:**
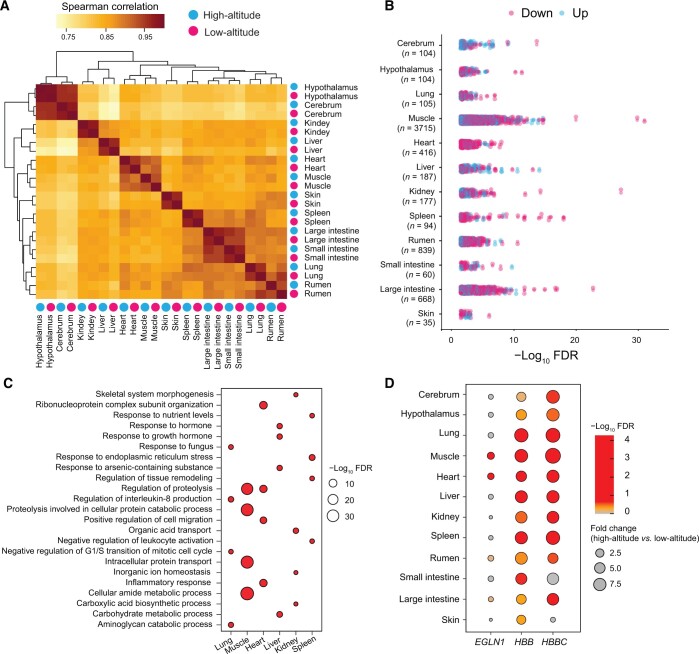
Global gene expression in 12 organs between the high-altitude and low-altitude sheep **A**. Clustering of samples was based on whole-genome expression values calculated as the TPM. The correlation between samples was measured by Spearman’s rank correlation coefficient. **B**. A schematic view of the DEGs in 12 organs. **C**. The enriched GO biological processes in cardiovascular and musculoskeletal systems. **D**. Global expression of three protein-coding genes with differential expression in at least one organ. TPM, transcripts per million; DEG, differentially expressed gene; GO, Gene Ontology; FDR, false discovery rate.

We investigated transcriptomic data from 12 organs (cerebrum, hypothalamus, lung, muscle, heart, liver, kidney, spleen, rumen, small intestine, large intestine, and skin) from high-altitude individuals [*n* = 4, Tibetan sheep at 4470 m above sea level (a.s.l.)] and low-altitude individuals (*n* = 4, Hu sheep at 400 m a.s.l.) to identify differentially expressed genes (DEGs). Organ-specific gene regulation was apparent in six organs (heart, liver, spleen, lung, kidney, and muscle) (**Figure 3**A). There were 4903 DEGs (ranging from 35 in the skin to 3715 in the muscle) between the high-altitude and low-altitude sheep (Figure 3B; Table S5). These DEGs were clustered together in organs with similar functions. Specifically, in the cerebral and hypothalamic tissues, DEGs were predominantly associated with Gene Ontology (GO) terms pertinent to neural functions. Concurrently, in the spleen and skin tissues, the enrichment was predominantly observed within immune-responsive terms. Furthermore, the rumen, large intestine, small intestine, and liver displayed a preponderance of DEGs associated with digestive system-related processes (Table S6).

Significant GO terms of the DEGs in six energy-demanding organs (heart, liver, spleen, lung, kidney, and muscle) were involved in responses to the movement process (“skeletal system morphogenesis”, “response to hormone”, “regulation of tissue remodeling”, and “inorganic ion homeostasis”), metabolic process (“response to nutrient levels”, “regulation of proteolysis”, “organic acid transport”, “carbohydrate metabolic process”, and “aminoglycan catabolic process”), and immune response (“regulation of interleukin-8 production”, “negative regulation of leukocyte activation”, and “inflammatory response”) (Figure 3C; Table S6).

**Figure 4:**
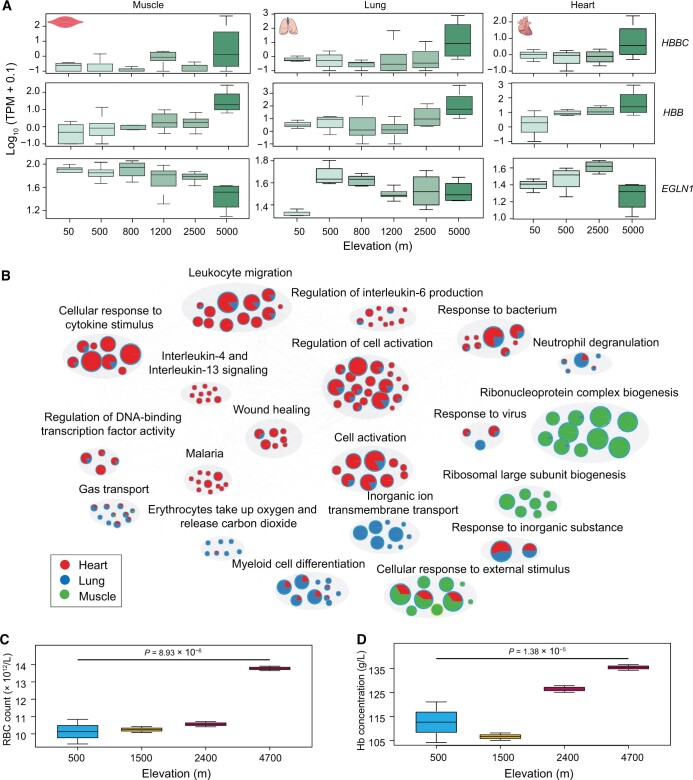
mRNA expression and biological functions of DEGs in the muscle, lung, and heart of sheep at different altitudes **A**. Transcription patterns of *HBBC*, *HBB*, and *EGLN1* in the muscle, lung, and heart of sheep at six different altitudes. **B**. The association between mRNA expression of candidate genes and altitudes (from 0 to 5000 m a.s.l.) was tested with the R lm() function. Gene sets within the top 100 genes and a *P* value less than 0.05 in three organs were selected for GO enrichment analysis. Gray circles represent the clusters of gene sets enriched for genes differentially expressed in heart (red), lung (blue), and muscle (green). Balloon size corresponds to the percentage of genes under GO terms from the corresponding gene list. **C**. and **D**. Comparison of RBC count (C) and Hb concentration (D) in sheep residing at varying altitudes. *P* values were determined using the Student’s *t*-test. RBC, red blood cell; Hb, hemoglobin; mRNA, messenger RNA; a.s.l., above sea level.

Three (*HBBC*, *HBB*, and *EGLN1*) of the ten genes within the adaptation-associated genomic regions were differentially transcribed (adjusted *P* < 0.05) in at least one organ. The difference in transcript abundance between the high-altitude and low-altitude sheep was pronounced for *HBBC* and *HBB* (Figure 3D). We further investigated the transcription patterns of these three genes in previously published transcriptomic data of sheep raised at variable altitudes up to 5000 m a.s.l. (https://www.ncbi.nlm.nih.gov/sra/?term=TIBET+SHEEP) [43]. These data included 158, 35, and 15 transcriptomes from muscle, lung, and heart, respectively. We found that *HBBC* and *HBB* were highly transcribed in the muscle (*HBB*: *P* = 1.09 × 10^−6^; *HBBC*: *P* = 7.23 × 10^−4^), lung (*HBB*: *P* = 1.62 × 10^−5^; *HBBC*: *P* = 4.24 × 10^−4^), and heart (*HBB*: *P* = 2.39 × 10^−3^; *HBBC*: *P* = 1.34 × 10^−3^) of the high-altitude sheep relative to the low-altitude sheep. On the other hand, the transcription of *EGLN1* was significantly lower in the muscle (*P* = 4.36 × 10^−4^) of the high-altitude sheep (**Figure 4**A). The GO analysis showed that the top 100 DEGs in the heart were enriched in “leukocyte migration”, “regulation of cell activation”, and “wound healing”; the top 100 DEGs in the lung were enriched in “gas transport”, “erythrocytes take up oxygen and release carbon dioxide”, and “inorganic ion transmembrane transport”; and the top 100 DEGs in the muscle were enriched in “cellular response to external stimulus”, “response to inorganic substance”, and “ribonucleoprotein complex biogenesis” (Figure 4B).

We collected fresh blood samples from adult sheep living at both high altitudes and low altitudes and measured their blood parameters within 12 h of collection. These records were combined with previously published data [44]. The results showed that Tibetan sheep residing at 4700 m a.s.l. had significantly higher red blood cell (RBC) count (*P* = 8.93 × 10^−6^) and Hb concentration (*P* = 1.38 × 10^−5^) compared with sheep residing at 500 m a.s.l. (Figure 4C and D). This suggests that Tibetan sheep augment their oxygen transport capacity by increasing both their RBCs and Hb levels to achieve an enhanced adaptability to high-altitude environments. This observation is consistent with the blood parameters that we previously reported in goats [45].

### Mapping potentially functional variants at the β-globin locus

**Figure 5:**
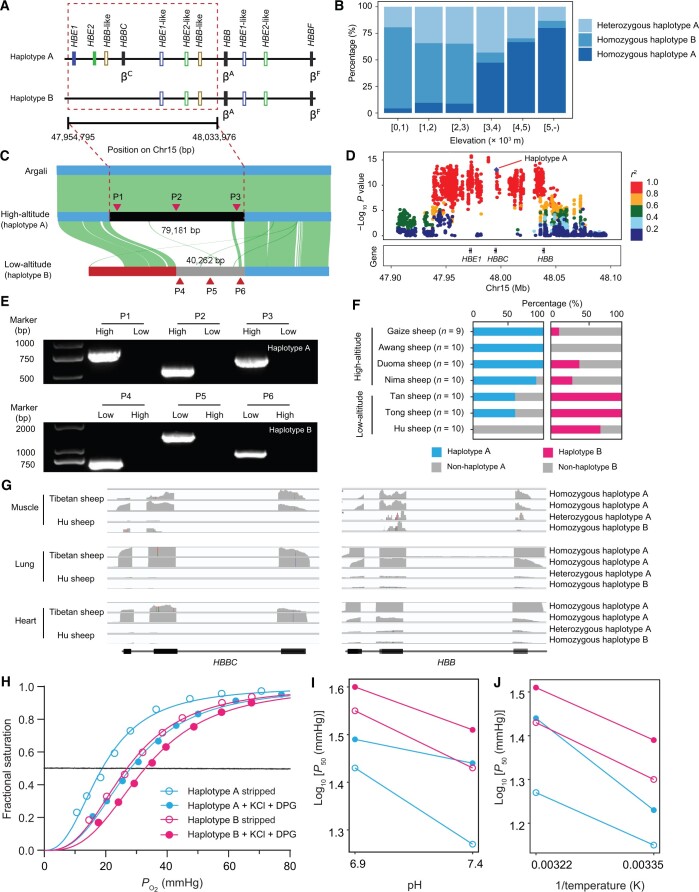
Association of SVs at the **β**-globin locus with different altitudes **A**. A schematic diagram of the sheep β-globin locus. The specific SVs are between haplotypes A and B, where haplotype B possesses an approximately 40-kb deletion. **B**. The frequency distribution of haplotypes A and B in sheep at different altitudes. **C**. Genomic alignment of the β-globin locus in argali, high-altitude (Tibetan), and low-altitude (Texel) sheep. Red triangles indicate primer positions. **D**. The zoom-in GWAS signals on Chr15. LD between the SV (the diamond-shaped blue point) and other variants (SNPs and INDELs < 50 bp) is quantified by the squared Pearson coefficient (*r*^2^). **E**. PCR-based genotyping verified the haplotypes A and B in the high-altitude (High) and low-altitude (Low) sheep. **F**. Frequency of the haplotypes A and B in the high-altitude and low-altitude sheep. **G**. IGV visualization of the mRNA expression between the haplotypes A and B in three organs (muscle, lung, and heart) of Tibetan and Hu sheep. **H**. The O_2_ equilibrium curves of haplotypes A and B Hbs in the absence (stripped) or presence of 0.1 M KCl and 0.2 mM DPG at 37°C and pH 7.4. **I**. Bohr effect of haplotypes A and B Hbs at 37°C, as indicated by a plot of log_10_*P*_50_*vs.* pH in the absence (stripped) or presence of 0.1 M KCl and 0.2 mM DPG. Bohr factor (*Φ*) is equal to the slope of the corresponding linear plot. **J**. Temperature effects (reflected by the overall change of enthalpy for oxygenation Δ*H′*) on the O_2_ affinity of haplotypes A and B Hbs at pH 7.4, as indicated by a plot of log_10_*P*_50_*vs.* 1/temperature (K) in the absence (stripped) or presence of 0.1 M KCl and 0.2 mM DPG. SV, structural variant; GWAS, genome-wide association study; LD, linkage disequilibrium; PCR, polymerase chain reaction; IGV, Integrative Genomics Viewer; DPG, 2,3-diphosphoglycerate.

According to the genomic structure at the β-globin locus, domestic sheep were divided into two groups based on the two haplotypes A and B [41]. We examined structural variants (SVs) at this locus using the PacBio HiFi data from 13 sheep representing different breeds [46], and found that Tibetan, Yunnan, Merino, East Friesian, Ujumqin, and Charollais sheep carried the haplotype A (**Figure 5**A), while Kazak, Dorset, Romney, Suffolk, Texel, White Dorper, and Kermani sheep carried the haplotype B (Figure 5A, Figure S4). These findings confirmed that the sheep haplotypes A and B at the β-globin locus were highly diverged, indicating their rather ancient evolutionary origins and likely unique phylogeographic patterns among different sheep breeds/populations [41].

Subsequently, we used short-read sequencing data of 924 domestic sheep to study the distribution pattern of haplotype A among worldwide populations inhabiting different altitudes. The examination of short-read alignments enabled us to differentiate between the three diplotypes as (1) homozygous haplotype A with high coverage, (2) heterozygous haplotype A with low coverage in the ∼ 40-kb region, and (3) homozygous haplotype B with the large ∼ 40-kb deletion (Figure S5). The distribution of these three diplotypes showed that the frequency of haplotype A increased with altitudes. For instance, its frequency was 0.93 in 98 high-altitude sheep (inhabiting areas around 5000 m a.s.l.), while it was 0.24 in 422 low-altitude sheep (inhabiting areas lower than 1000 m a.s.l.) (Figure 5B). Interestingly, the haplotype A was fixed in argali and dominant in European (f_AA_ = 0.36; f_AB_ = 0.41; f_BB_ = 0.23) and Asiatic (f_AA_ = 0.39; f_AB_ = 0.5; f_BB_ = 0.11) mouflons, while it was almost absent in other wild sheep species (Figures S6 and S7). Pairwise sequence alignment identified a structurally homologous sheep haplotype A fragment in the argali genome, verifying its ancient origin before their speciation (Figure 5C). The 787 significant SNPs and INDELs within the haplotype A fragment were in strong linkage disequilibrium (LD, *r*^2^ > 0.8), suggesting that this fragment has conferred the association signal on chromosome 15 (Figure 5D). Indeed, the signal was absent when the association analysis was conditional on haplotype A (Figure S8). We then evaluated the distribution of haplotypes A and B in additional 69 sheep representing seven breeds (high-altitude: Gaize, Awang, Duoma, and Nima; low-altitude: Tan, Tong, and Hu) using polymerase chain reaction (PCR) amplification (Figure 5E and F; Tables S7–S9). The result confirmed that the haplotype A was dominant in high-altitude sheep, whereas the haplotype B was predominant in low-altitude sheep. Notably, by linking the expression of specific haplotypes of the candidate genes, the transcriptomic data showed that both *HBBC* and *HBB* were highly transcribed in the muscle, lung, and heart of the high-altitude haplotype A carriers, while the transcription of *HBB* was relatively low in the low-altitude haplotype B carriers (Figure 5G).

The O_2_ affinity of purified Hbs plays a critical role in realizing the high-altitude adaptability of animals. To determine how blood–O_2_ affinity could be affected by the haplotypes A and B, we measured haplotype-specific O_2_-binding properties in purified Hbs. Under different experimental conditions [37°C, pH = 7.4, and the presence or absence of chloride ions (KCl) and organic phosphates 2,3-diphosphoglycera (DPG)], the *P*_50_ of the haplotype A Hbs was significantly smaller than that of the haplotype B (*P* < 1.50 × 10^−4^), indicating that the haplotype A had a higher intrinsic O_2_ affinity (Figure 5H; Table S10). With the changes in both pH and temperature, the *P*_50_ values of the haplotypes A and B exhibited broadly similar downward trends (Figure 5I and J). These findings suggest that sheep carrying the high-altitude haplotype A have a significantly enhanced O_2_ affinity compared with those carrying the low-altitude haplotype B.

### Mapping potentially functional variants in the *EGLN1* region

**Figure 6:**
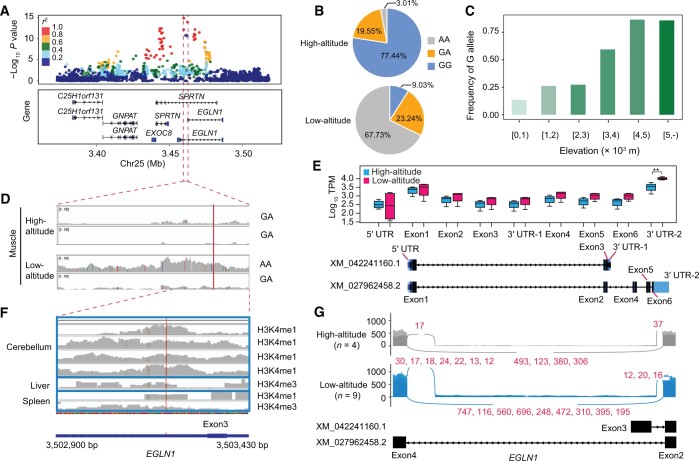
Association of the *EGLN1* G/A SNP (Chr25:3,503,284 bp) with different altitudes **A**. The zoom-in GWAS signals on Chr25. LD between the G/A SNP and other variants is quantified by the squared Pearson coefficient (*r*^2^). **B**. Allele frequency of the G/A SNP between the high-altitude and low-altitude sheep. **C**. Allele frequency of the putatively adaptive G allele in worldwide sheep populations. **D**. The expression level of mRNA in the G/A SNP region and its corresponding genotypes. **E**. The exonic expression of *EGLN1* in the muscle between the high-altitude and low-altitude sheep. **F**. The epigenetic signals in the region encompassing the putatively adaptive G allele. **G**. Splice junction patterns between exons 2 and 4. The two transcripts of *EGLN1* at the corresponding positions are shown at the bottom. The numbers in red indicate the reads shared between the two transcripts; each number corresponds to a sample, and the numbers are separated by commas. UTR, untranslated region.

*EGLN1* (also known as HIF prolyl hydroxylase domain 2, *PHD2*) spans ∼ 49.4 kb and contains six exons. We detected 23 variants within the *EGLN1* region to be significantly associated with altitudes, including a G/A SNP (Chr25:3,503,284 bp; *P* = 1.75 × 10^−11^) in its 3′ untranslated region (3′ UTR). This SNP was in strong LD (*r*^2^ > 0.8) with 22 surrounding SNPs and INDELs (**Figure 6**A). The allele frequencies of this SNP differed between the Tibetan sheep and low-altitude populations. Up to 77.44% of the Tibetan sheep carried the GG genotype, while only 9.03% of low-altitude sheep had the GG genotype (Figure 6B). The frequency of the G allele was positively correlated with altitudes among the Tibetan sheep and other populations (*r*^2^ = 0.92) (Figure 6C, Figure S9). The transcriptomic data of the muscle demonstrated that the 3′ UTR and all exonic regions of *EGLN1* were down-regulated in high-altitude individuals (Figure 6D and E). We next annotated all 23 significantly associated variants using epigenomic data [47] and found that the G/A SNP was located within a H3K4me1 peak (Figure 6F; Table S4). We further found that an *EGLN1* alternative splicing in the 3′ UTR region likely led to an exon elongation in one of the *EGLN1* transcripts, XM_042241160.1 (Figure 6G, Figures S10 and S11). Interestingly, the GC content in the first promoter exon of *EGLN1* was as high as 82.2%. It is known that GC-rich sequences are challenging to sequencing [48,49], but the coverage of mapped DNA reads in this region was surprisingly higher in the high-altitude than in the low-altitude sheep (Figure S12). This suggests that this GC-rich region of the promoter may regulate the activity of *EGLN1*. However, further investigations are required to support such a claim.

## Discussion

In this study, we conducted a large-scale genome-wide association scan to elucidate the genetic basis of high-altitude adaptation in sheep. By integrating the large-scale genome-wide association study (GWAS) data with the transcriptomes of 12 organs isolated from high-altitude and low-altitude sheep, we investigated a genomic landscape of high-altitude genetic determinism and highlighted three candidate genes (*HBBC*, *HBB*, and *EGLN1*) and two haplotypes A and B at the β-globin locus associated with high-altitude adaptation in Tibetan sheep.

Along with our recent study that revealed the high-altitude adaptability of Tibetan goats [28], several reports have shown that beneficial alleles facilitated a better fitness of the local dwellers on the QTP. Examples of the adaptive alleles include high-altitude dogs [16,17,50,51], yaks [31], and Tibetan cattle [52,53]. In humans, substantially advantageous alleles enabled Tibetans to be better adapted to environmental challenges such as hypoxia and UV radiation [11–13,30]. The *HBB* locus in Tibetan sheep was previously thought to have been introgressed from argali [4]. However, the genomic region (Chr15:47,400,001–47,500,000 bp) in the Oar_v4.0 (GCA_000298735.2) from a Texel sheep corresponded to the haplotype B [41], in which only *HBE* and *HBB* were annotated [4]. This region was nevertheless recaptured in the Changthangi sheep distributed at high altitude and the variants of its *HBB* and/or neighboring genes were shown as the result of wild introgression, while *HBB*, *HBB*-like, *HBBC*, and *HBE2* were found to have differentiated as putatively selected genes, based on an early sheep reference genome Oar_rambouillet_v1.0 (GCF_002742125.1) [32]. Compared with the complete genomic sequence of the β-globin locus in the latest ARS-UI_Ramb_v2.0 (NC_056068.1:47,967,454–48,084,335) [39], the homologous β-globin locus in the Oar_rambouillet_v1.0 (NC_040266.1:51,913,702–52,096,364) had an extra 66-kb redundant sequence, indicating its poor assembly.

In this study, we utilized the PacBio HiFi reads of 13 domestic sheep [46] and the genome of argali sheep [54] to examine the detailed genomic structure of the β-globin locus, at which the Tibetan sheep and argali carried the haplotype A with highly homologous genomic and gene structures in the ARS-UI_Ramb_v2.0. PCR genotyping of 69 sheep samples validated the dominant distribution of haplotype A in Tibetan sheep, while the genotypes at the β-globin locus of 924 domestic sheep identified a genetic cline of increasing haplotype A alongside the elevated altitudes. Previous studies have indicated that the β-globin locus in sheep contains several SNPs originating from argali through introgression [4]. We also confirmed an extensively phylogeographic distribution of haplotype A in argali, European and Asiatic mouflons, and domestic sheep breeds/populations [41]. All these findings strongly supported ancient and independent evolutionary origins of haplotypes A and B at the β-globin locus in sheep [41], while the haplotype A has been becoming dominant in Tibetan sheep as a result of strong natural selection driving their rapid adaptation to high-altitude challenges. Therefore, we postulated that the haplotype A was likely segregated in the ancestral population of Tibetan sheep before their migration to the QTP. Upon their arrival, the natural selection favoring the haplotype A, in conjunction with the adaptive introgression from argali, may have expedited the fixation of haplotype A within the Tibetan sheep, enabling their rapid adaptation to the high-altitude environment. Moreover, the differential expression of both *HBBC* and *HBB* in the muscle, lung, and heart between high-altitude and low-altitude sheep provided solid evidence that the functional β-globin genes embedded in the haplotype A increased the oxygen transportation of Tibetan sheep to cope with the hypoxic conditions on the QTP [55–57]. Additionally, sheep carrying haplotype A exhibited an enhanced Hb–O_2_ affinity, suggesting a more effective bindability to oxygen under hypoxic conditions. In contrast, those with the low-altitude haplotype B had a relatively low Hb–O_2_ affinity, allowing their survival in the areas with ample oxygen supply.

We also detected an adaptive allele at the second strongest locus around the *EGLN1* gene, which showed differential expression in muscle between the high-altitude and low-altitude sheep, suggesting its contribution to the adaptation of Tibetan sheep to high-altitude environments. *EGLN1* was also found to be associated with high-altitude adaptation in humans [11,42], cattle [53], and ducks [58], indicating its convergent evolution across different species. *EGLN1* has been reported to be a regulator of *EPAS1*, which directly regulates the expression of erythropoietin (EPO) and eventually influences the Hb level in the blood [42]. Under the hypoxic challenge, hydroxylation significantly decreases, and *EPAS1* and HIF1α are stabilized, leading to an improved hypoxia adaptation [59]. Although a previous study indicated that *EPAS1* was under selection in Tibetan sheep [33], the results of all three methods (GWAS, XP-EHH, and XP-CLR) applied in this study did not recapture any signals of positive selection for *EPAS1*. Given these observations, we assumed that varying population differences may account for the discrepancies across genome scans. Similar to particular morphological traits such as horn size and shape [4,37], the beneficial allele of *EGLN1* was likely involved in the adaptive evolution of Tibetan sheep to withstand harsh environments on the QTP.

## Conclusion

In summary, based on the integrating analysis of short-read sequencing, PacBio HiFi, and transcriptomic data together with the Hb–O_2_ affinity of sheep at different altitudes, our results validated that haplotype A was associated with hypoxic adaptation, and further confirmed the ancient and independent evolutionary origins of haplotypes A and B at the β-globin locus. We also detected a new gene, *EGLN1*, associated with high-altitude adaptability in Tibetan sheep. These data and findings provide valuable resources for livestock breeding and contribute to the understanding of high-altitude diseases in humans.

## Materials and methods

### Sample collection and DNA sequencing

Blood samples from 77 sheep were collected from different geographic locations: 64 from high-altitude Nima, Kangma, Gaize, Gangba, Duoma, and Awang of Xizang Autonomous Region (> 3000 m a.s.l.), 10 from Lanping, Yunnan Province (∼ 1600 m a.s.l.), two from low-altitude Yangling, Shaanxi Province (< 1000 m a.s.l.), and one argali sampled at the Beijing Zoo, Beijing, China. DNA was isolated from whole blood samples using the DNeasy Blood and Tissue Kit (Catalog No. 69506, Qiagen, Dusseldorf, Germany). Paired-end sequencing libraries were prepared using the Genomic DNA Sample Prep Kit (Catalog No. 20060060, Illumina, San Diego, CA). Libraries were sequenced using the Illumina Sequencing Kit v3 (Catalog No. MS-102-3001, Illumina) on an HiSeq 2500 (Catalog No. Hiseq 2500, Illumina) instrument at Novogene (http://cn.novogene.com).

### Read alignment and variant calling

Trimmomatic (v0.36) [60] was used for quality control and adapter trimming before reads were mapped to the domestic sheep reference genome (ARS-UI_Ramb_v2.0, GCA_016772045.1) [39] using BWA-MEM (v0.7.15) [61]. The aligned Binary Alignment/Map format (BAM) files were coordinate-sorted, and read groups were merged into the same sample using SortSam and MergeSamFiles from the Picard tools software suite (v2.1; https://broadinstitute.github.io/picard/). Duplicates were also marked using the Picard tools software suite (v2.1; https://broadinstitute.github.io/picard/). The coverage at each called site was estimated for each sample using Qualimap (v2.2) [62]. Genotype calling was performed using HaplotypeCaller and GenotypeGVCFs from the GATK (v4.1.7.0) [40]. The VariantFiltration module of GATK was used to remove SNPs if QD < 2.0, FS > 60.0, MQRankSum < −12.5, ReadPosRankSum < −8.0, SOR > 3.0, and MQ < 40.0. INDELs up to 50 bp were also retained and filtered using maxIndelSize = 50, QD < 2.0, FS > 200.0, and ReadPosRankSum < −20.0.

### Population genetic analysis

PLINK (v1.9) [63] was used to retain biallelic SNPs that had a minor allele frequency (MAF) higher than 0.05 and did not show an excess of heterozygosity (--hwe 0.001). We excluded SNPs in LD with *r*^2^ > 0.2 (--indep-pairwise 50 5 0.2). These filters retained 4,899,522 high-quality SNPs. A PCA was performed with the smartpca program from the EIGENSOFT package [64] with default parameters and the following settings: numoutlieriter = 0 and numchrom = 26.

### Association tests

To exclude the effect of tail size, we only used thin-tailed sheep similar to Tibetan sheep for altitude association analysis and obtained a set of 450 individuals from various populations and altitudes. BCFtools [65] was used to extract 450 individuals from the total unfiltered Variant Call Format (VCF) file, and PLINK (v1.9) [63] was used to extract 34,298,967 variants (31,567,131 SNPs and 2,731,836 INDELs) with MAF higher than 0.05. Association analysis was conducted with the genome-wide efficient mixed-model association (GEMMA) software [66]. The Bonferroni-corrected significance threshold was applied to identify significantly associated variants, using the univariate linear mixed model, which is a general and widely used model with the following formula:

$$y=W\alpha \;+\;x\beta \;+\;u\;+\;\epsilon ;\;u∼{\text{MVN}}_{n}\left(0,\;\lambda \tau^{-1}K\right),\epsilon ∼{\text{MVN}}_{n}\left(0,\tau^{-1}I_{n}\right)$$

where ***y*** is an *n*-vector of quantitative traits (or binary disease labels) for *n* individuals; **W** = (**w**_1_, ···, **w**_*c*_) is an *n* × *c* matrix of covariates (fixed effects) including a column of 1s (intercept); ***α*** is a *c*-vector of the corresponding coefficients including the intercept; ***x*** is an *n*-vector of marker genotypes; *β* is the effect size of the marker; ***u*** is an *n*-vector of random effects; ***ϵ*** is an *n*-vector of errors; *τ*^−1^ is the variance of the residual errors; λ is the ratio between the two variance components; **K** is a known *n* × *n* relatedness matrix; **I**_*n*_ is an *n* × *n* identity matrix; and MVN*n* denotes the *n*-dimensional multivariate normal distribution. We adjusted for potential population stratification using the first three principal components. To reduce the likelihood of false positive signals, we set the genome-wide significance threshold to 1.46 × 10^−9^. This value corresponds to a *P* value of 0.05 divided by the total number of SNPs and INDELs, as determined by the Bonferroni correction.

### Identification of selection signatures

We applied XP-EHH [67] and XP-CLR [68] analyses and used data from 145 Tibetan sheep (altitude > 3000 m a.s.l.) and 119 low-altitude sheep (altitude < 1200 m a.s.l.). These estimates were determined using 50-kb window that slides in 10-kb increments across the genomes. The threshold value corresponds to the top 1% of the XP-EHH values (XP-EHH < −1.74 or XP-EHH > 1.99) and XP-CLR values (−log_10_ XP-CLR > 1.943).

### RNA sequencing

Total RNA of 12 organs (cerebrum, hypothalamus, lung, muscle, heart, liver, kidney, spleen, rumen, small intestine, large intestine, and skin) from 8 adult sheep (*n* = 4 Tibetan sheep raised at high altitudes and *n* = 4 Hu sheep raised at low altitudes) was extracted using the TRIzol reagent (Catalog No. 15596026CN, Thermo Fisher Scientific, Waltham, MA) according to the manufacturer’s instructions. The RNA samples were subjected to quality control using a NanoDrop 2000 spectrophotometer (Catalog No. ND2000, Thermo Fisher Scientific), agarose gel electrophoresis, and the Agilent 2100 Bioanalyzer (Catalog No. 2001, Agilent Technologies, Santa Clara, CA). The RNA sequencing (RNA-seq) library construction and paired-end sequencing were done at Novogene according to the company’s pipeline. The average number of reads per sample was 30,195,473.

From the raw RNA-seq data, adapter sequences, reads with more than 5% unknown nucleotides, and reads with many low-quality bases (more than half of the bases’ qualities were less than 10) were removed with Trimmomatic (v0.36) [60]. Transcript abundance was quantified in transcripts per million (TPM) using kallisto (v0.46.1; https://pachterlab.github.io/kallisto) [69] with default parameters and an index built from the GCF_016772045.1_ARS-UI_Ramb_v2.0_rna.gz from Ensembl. Transcript abundance was aggregated to the gene level using the tximport package [70] of the R software. The edgeR package [71] was used to calculate the differential expression of genes with a minimum fold change of 1 and a false discovery rate (FDR) lower than 0.05. GO term annotations were performed using Metascape [72]. The association between messenger RNA (mRNA) expression of candidate genes and altitude (from 0 to 5000 m a.s.l.) was tested with the R lm() function. Gene sets with the top 100 genes and a *P* value less than 0.05 were selected for GO term analysis. We utilized all genes in the genome as the enrichment background. Terms that met the following criteria were gathered and clustered based on their membership similarities: a *P* value less than 0.01, a minimum count of 3, and an enrichment factor greater than 1.5 (the enrichment factor is the ratio between the observed counts and the counts expected by chance). *P* values were calculated using the cumulative hypergeometric distribution. Exon-specific mRNA expression was quantified using QTLtools [73].

### Determination of SVs

SVs in candidate regions were investigated using the Integrative Genomics Viewer (IGV) in PacBio HiFi sequencing data [74]. The mosdepth software (v0.2.2) [75] was used to calculate the sequencing coverage within candidate regions. The coverage at the candidate region (Chr15:47,954,795–48,033,976 bp) was extracted and divided by the average coverage of chromosome 15 to differentiate between haplotypes A and B. The diplotypes were defined as follows: haplotype A frequency ≥ 0.75, homozygous haplotype A; 0.25 ≤ haplotype A frequency < 0.75, heterozygous haplotype A; and 0 ≤ haplotype A frequency < 0.25, homozygous haplotype B. Pairwise linear alignments between the genomes of different breeds and species at different altitudes were performed using the minimap2 software [76]. The genomes of argali (JAKZEL000000000.1) [54], the high-altitude Qiaoke sheep (ASM2241668v1, GCA_022416685.1) [46], and the low-altitude Texel sheep (Oar_v4.0, GCA_000298735.2) [77] were used to investigate SVs.

### Validation of SVs using PCR amplification

Genomic DNA was extracted from blood samples using a Blood Genomic DNA Mini Kit (Catalog No. CW2087S, CWBIO, Taizhou, China). Then, the extracted DNA was used for PCR-based genotyping. Six pairs of primers were designed to amplify regions located upstream, downstream, and within the deletion region. A thermostable Taq DNA polymerase (Catalog No. 10106ES03, Yeasen, Shanghai, China) was used for the PCR reaction, and the following PCR amplification program was used for all primer pairs: initial denaturation at 94°C for 3 min; 36 cycles of 94°C for 10 s, 58°C for 20 s, and 72°C for 1 min; and 72°C for 5 min. Amplified fragments were evaluated on a 1.5% agarose gel. Briefly, successful amplification of PCR products indicates no deletion, whereas failed amplification from the sample indicates deletion. Primer sequences used are listed in Table S7.

### Blood parameter analysis

Fresh blood samples (5 ml) were collected from 10 adult Dorset sheep living at low altitudes and measured within 12 h of collection for their hematological profiles, including RBC count and Hb concentration, which were combined with previously published data of 215 adult sheep living at both high and low altitudes [44].

### Functional analysis of total Hbs in blood

Blood samples (∼ 200 μl) were added to a 5× volume of ice-cold water and incubated on ice to lyse the RBCs for 30 min. The samples were then centrifuged at 20,000 *g* for 10 min to remove cell debris. Buffer was added to the supernatants to a final concentration of 0.01 M 4-(2-hydroxyethyl)-1-piperazine ethanesulfonic acid (HEPES)/0.2 M NaCl (pH 7.4), and passed through a PD-10 desalting column (Catalog No. 28-9493-23, GE Healthcare, Chicago, IL) equilibrated with 25 ml of 0.01 M HEPES (pH 7.4) to remove intracellular cofactors.

Hb solutions [0.1 mM Hb in 0.1 M HEPES/0.05 M ethylene diamine tetraacetic acid (EDTA) buffer (pH 7.4)] used for O_2_ equilibrium measurement were freshly prepared in the absence (stripped) or presence of 0.1 M KCl and 0.2 mM DPG [78,79]. O_2_ equilibrium curves were measured using a modified diffusion chamber method as previously described [80,81]. Curves were measured in 0.1 M HEPES buffer with pH 6.9 or pH 7.4 at 37°C to estimate the Bohr effect, and with pH 7.4 at 25°C to estimate the enthalpy of oxygenation.

Hill plots {log_10_ [fractional saturation/(1 − fractional saturation)] *vs.* log_10_$P_{O_{2}}$} constructed from these measurements were used to estimate the partial pressure of oxygen ($P_{O_{2}}$) at half saturation (*P*_50_) and the cooperativity coefficient (*n*_50_) from the χ-intercept and slope of these plots, respectively. O_2_ equilibrium curves for Hb solutions were measured four times, whereas *P*_50_ was recoded as mean ± standard error of mean (SEM) [24,51,82].

The Bohr factor was quantified by the slope of linear plots of log_10_*P*_50_ as a function of pH in the range of pH 6.9 and pH 7.4 (*Φ* = Δlog_10_*P*_50_/ΔpH). The overall change of enthalpy for oxygenation at pH 7.4 was calculated by using the Van’t Hoff Equation Δ*H* = 2.303R [Δlog_10_*P*_50_/(Δ1/T)], where R is the gas constant (8.314 J/mol/K) and T is the absolute temperature in Kelvin [83]. The Δ*H′* values were corrected by excluding the heat of O_2_ in solution (−12.6 kJ/mol O_2_) [84].

## Ethical statement

The present study was approved by the Animal Care and Use Committee of Northwest A&F University (Approval No. 2020DK0802). All methods were carried out in accordance with relevant guidelines and regulations of Northwest A&F University, China.

## Supplementary Material

qzae030_Supplementary_Data

## Data Availability

The raw sequence data reported in this study have been deposited in the Genome Sequence Archive [85] at the National Genomics Data Center [86], Beijing Institute of Genomics, Chinese Academy of Sciences / China National Center for Bioinformation (GSA: CRA011038), and are publicly accessible at https://ngdc.cncb.ac.cn/gsa.

## CRediT author statement

**Chao Li:** Data curation, Formal analysis, Investigation, Resources, Writing – original draft, Writing – review & editing. **Bingchun Chen:** Investigation, Validation. **Suo Langda:** Resources, Validation. **Peng Pu:** Validation. **Xiaojia Zhu:** Investigation, Writing – review & editing. **Shiwei Zhou:** Resources. **Peter Kalds:** Writing – original draft, Writing – review & editing. **Ke Zhang:** Resources. **Meenu Bhati:** Formal analysis. **Alexander Leonard:** Formal analysis. **Shuhong Huang:** Formal analysis. **Ran Li:** Formal analysis, Investigation. **Awang Cuoji:** Resources. **Xiran Wang:** Validation. **Haolin Zhu:** Validation. **Yujiang Wu:** Conceptualization, Resources, Funding acquisition. **Renqin Cuomu:** Resources. **Ba Gui:** Resources. **Ming Li:** Formal analysis, Investigation. **Yutao Wang:** Resources. **Yan Li:** Resources. **Wenwen Fang:** Formal analysis. **Ting Jia:** Resources. **Tianchun Pu:** Resources. **Xiangyu Pan:** Formal analysis. **Yudong Cai:** Formal analysis. **Chong He:** Formal analysis. **Liming Wang:** Resources. **Yu Jiang:** Investigation. **Jian-Lin Han:** Conceptualization, Writing – review & editing. **Yulin Chen:** Conceptualization, Investigation, Funding acquisition. **Ping Zhou:** Conceptualization, Resources. **Hubert Pausch:** Conceptualization, Data curation, Formal analysis, Investigation, Supervision, Writing – review & editing. **Xiaolong Wang:** Conceptualization, Data curation, Formal analysis, Investigation, Resources, Supervision, Writing – review & editing, Funding acquisition. All authors have read and approved the final manuscript.

## ORCID

0000-0002-2769-7616 (Chao Li)

0009-0008-0908-3056 (Bingchun Chen)

0009-0008-9458-5906 (Suo Langda)

0000-0001-6960-3836 (Peng Pu)

0000-0001-5330-1278 (Xiaojia Zhu)

0009-0008-7732-9257 (Shiwei Zhou)

0000-0002-4276-483X (Peter Kalds)

0000-0002-2392-1545 (Ke Zhang)

0000-0003-4955-2255 (Meenu Bhati)

0000-0001-8425-5630 (Alexander Leonard)

0000-0003-2302-4200 (Shuhong Huang)

0000-0002-8584-4100 (Ran Li)

0009-0000-6736-1327 (Awang Cuoji)

0009-0008-4973-6963 (Xiran Wang)

0009-0000-6210-9026 (Haolin Zhu)

0000-0002-3915-4889 (Yujiang Wu)

0009-0002-2848-7511 (Renqin Cuomu)

0000-0002-8127-4809 (Ba Gui)

0000-0001-7049-8666 (Ming Li)

0000-0003-0580-5820 (Yutao Wang)

0000-0003-1446-7253 (Yan Li)

0000-0003-2016-2544 (Wenwen Fang)

0009-0005-8872-2650 (Ting Jia)

0009-0000-2485-6792 (Tianchun Pu)

0000-0001-6841-7652 (Xiangyu Pan)

0000-0001-5902-0901 (Yudong Cai)

0000-0001-5977-6629 (Chong He)

0000-0001-7088-3193 (Liming Wang)

0000-0003-4821-3585 (Yu Jiang)

0000-0002-1527-3963 (Jian-Lin Han)

0000-0001-5679-4055 (Yulin Chen)

0009-0007-3442-1232 (Ping Zhou)

0000-0002-0501-6760 (Hubert Pausch)

0000-0003-1620-1344 (Xiaolong Wang)
