# Supplementary material for: Multi-omic Analyses Shed Light on The Genetic Control of High-altitude Adaptation in Sheep
Source: Genomics Proteomics Bioinformatics. 2024 Apr 2;22(2):qzae030. doi: 10.1093/gpbjnl/qzae030 (PMC12016566; doi:10.1093/gpbjnl/qzae030)
Supplement: qzae030_Supplementary_Data [file qzae030_supplementary_data.zip › Figure S1.pdf]

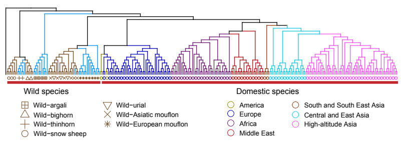
Wild species
Wild−argali
Wild−bighorn
Wild−thinhorn
Wild−snow sheep
Wild−urial
Wild−Asiatic mouflon
Wild−European mouflon
Domestic species
America
Europe
Africa
Middle East
South and South East Asia
Central and East Asia
High-altitude Asia
