# Supplementary material for: Multi-omic Analyses Shed Light on The Genetic Control of High-altitude Adaptation in Sheep
Source: Genomics Proteomics Bioinformatics. 2024 Apr 2;22(2):qzae030. doi: 10.1093/gpbjnl/qzae030 (PMC12016566; doi:10.1093/gpbjnl/qzae030)
Supplement: qzae030_Supplementary_Data [file qzae030_supplementary_data.zip › Figure S4.pdf]

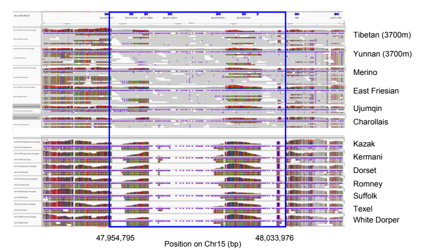
Tibetan (3700m)
Yunnan (3700m)
Merino
East Friesian
Ujumqin
Charollais
Kazak
Kermani
Dorset
Romney
Suffolk
Texel
White Dorper
47,954,795
48,033,976
Position on Chr15 (bp)
