# Supplementary material for: Multi-omic Analyses Shed Light on The Genetic Control of High-altitude Adaptation in Sheep
Source: Genomics Proteomics Bioinformatics. 2024 Apr 2;22(2):qzae030. doi: 10.1093/gpbjnl/qzae030 (PMC12016566; doi:10.1093/gpbjnl/qzae030)
Supplement: qzae030_Supplementary_Data [file qzae030_supplementary_data.zip › Supplementary material captions.docx]

**Supplementary material**

**Figure S1 NJ phylogenetic tree of 293 representative sheep based on Reynolds’ distances estimated using the whole genome-wide SNPs**

This includes both all wild species (*n* = 70) and 48 domestic breeds (*n* = 223). The wild individuals included 6 bighorn sheep, 6 thinhorn sheep, 11 argali, 16 Asiatic mouflons, 17 European mouflons, 6 urial, and 8 snow sheep. On the other hand, the domestic individuals included 4 from America, 46 from Europe, 43 from Africa, 23 from the Middle East, 4 from Southeast Asia, and 103 from Central Asia (including 39 newly sequenced data from Autonomous Region, China). NJ, neighbor-joining.

**Figure S2 Genetic diversity of sheep breeds included in this study**

PCA of sheep samples from Asia (**A**) and QTP of China (**B**) using whole-genome sequences. The 507 Asian sheep were from the geographical areas of South and Southeast Asia, Central and East Asia, Yunnan–Kweichow Plateau, and QTP. The 145 Tibetan sheep were sampled from the geographical areas of three provinces, namely Xizang Autonomous Region (Nima, Kangma, Gaize, Gangba, Duoma, Awang, Shigatse, Dangxiong, and Nyingchi), Qinghai (Huangnan), and Shaanxi (Hanzhong). PCA, principal component analysis; QTP, Qinghai-Tibet Plateau.

**Figure S3 Patterns of selected SNPs and INDELs (< 50 bp)**

Genotypic patterns of selected SNPs and INDELs (−log_10_ *P* value = 8.84) on chromosomes 3, 7, 9, 18, and 26 in 924 domestic sheep. The horizontal axis is the genomic region, while the vertical axis is the elevation.

**Figure S4 Detection of SVs in the *HBBC* region**

BAM file screenshots of PacBio HiFi data from 13 breeds (Tibetan, Yunnan, Merino, East Friesian, Ujumqin, Charollais, Kazak, Dorset, Romney, Suffolk, Texel, White Dorper, and Kermani sheep).

**Figure S5 Distribution of haplotype A**

The horizontal axis is the average read coverage of the chromosome, and the individual count corresponds to the vertical axis. The mosdepth software (v0.2.2) was used to extract the coverage of reads of the candidate region (Chr15:47,954,795–48,033,976 bp). The genotypes of haplotypes are defined as follows: haplotype A frequency ≥ 0.75, homozygous haplotype A; 0.25 ≤ haplotype A frequency < 0.75, heterozygous haplotype A; and 0 ≤ haplotype A frequency < 0.25, homozygous haplotype B.

**Figure S6 Distribution of haplotypes A and B in wild sheep from 69 published data and one individual argali newly sequenced in this study**

The haplotype detection method is the same as that indicated in Figure S5. BIH, Bighorn; THH, Thinhorn; SNOW, Snow sheep; VIG, Urial; AMU, Asiatic Mouflon; ORI, European Mouflon; ARG, Argali.

**Figure S7 Comparison of selected genomic regions identified in genome selection scans of wild sheep**

The haplotypic distribution of all selected SNPs and INDELs within the region Chr15:47,838,057–48,075,787 bp in seven wild sheep (BIH, THH, SNOW, VIG, AMU, ORI, and ARG).

**Figure S8 Manhattan plot representing the association of SNPs and INDELs with habitat altitudes in sheep (*n* = 450)**

The association analysis was conditional on the haplotype A and haplotype B status, with the haplotype (coded as 0, 1, and 2 for AA, AB, and BB, respectively) as a covariate. The dotted horizontal line indicates the Bonferroni-corrected significance threshold (−log_10_ *P* value = 8.84).

**Figure S9 Correlation of the G allele frequencies with the altitudes of different sheep populations**

The frequency of the G allele in different sheep populations was counted, and the ggplot2 package of the R language was used to perform the linear fit analysis between the G allele frequencies and altitudes.

**Figure S10 Alternative splicing of the *EGLN1* gene in the muscle of the high-altitude and low-altitude sheep**

The read distribution between exons 2 and 4 of *EGLN1* in the muscle of 13 individuals, of which 8 were newly sequenced and 5 were downloaded (<https://www.ncbi.nlm.nih.gov/sra/?term=TIBET+SHEEP>).

**Figure S11 BAM file screenshots of the *EGLN1* gene in transcriptomic data of the high-altitude and low-altitude sheep**

BAM file screenshots between exons 2 and 4 of *EGLN1* in the muscle tissues of 13 individuals.

**Figure S12 BAM file screenshots of the *EGLN1* gene in genome data of the high-altitude and low-altitude sheep**

The read coverages of *EGLN1* exon 1 between the high-altitude and low-altitude individuals (*n* = 10).

**Table S1 Summary of the information of 77 sheep newly sequenced in this study**

**Table S2 Summary of the information of 994 sheep genome data used in this study**

**Table S3 Sampling information of 293 individuals used in phylogenetic analysis**

**Table S4 Significant SNPs and INDELs identified based on GWAS**

**Table S5 DEGs in 12 organs**

**Table S6 The biological processes of DEGs in 12 organs**

**Table S7 Primers were used in this study**

**Table S8 The genotypic frequency of the haplotype A in each sheep population**

**Table S9 The genotypic frequency of the haplotype B in each sheep population**

**Table S10 O_2_ affinity (*P*_50_, mmHg), Hill’s cooperativity coefficients (n50), and Bohr factors of haplotypes A and B Hbs**
