# Supplementary figures and images for: Multi-omic Analyses Shed Light on The Genetic Control of High-altitude Adaptation in Sheep
Source: Genomics Proteomics Bioinformatics. 2024 Apr 2;22(2):qzae030. doi: 10.1093/gpbjnl/qzae030 (PMC12016566; doi:10.1093/gpbjnl/qzae030)

**A**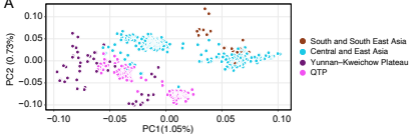**B**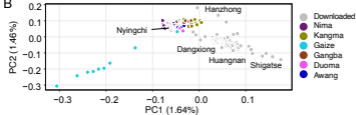

Supplement: qzae030_Supplementary_Data [file qzae030_supplementary_data.zip › Figure S2.pdf]

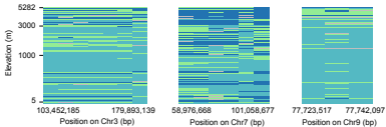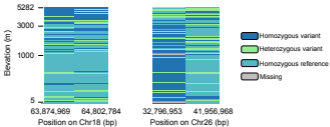

Supplement: qzae030_Supplementary_Data [file qzae030_supplementary_data.zip › Figure S3.pdf]

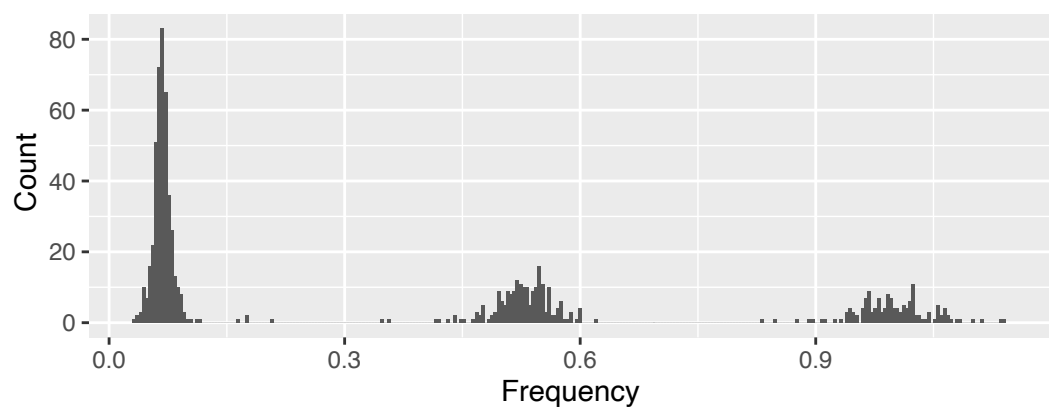

Supplement: qzae030_Supplementary_Data [file qzae030_supplementary_data.zip › Figure S5.pdf]

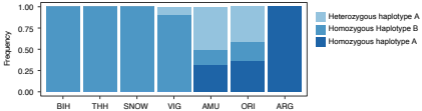

Supplement: qzae030_Supplementary_Data [file qzae030_supplementary_data.zip › Figure S6.pdf]

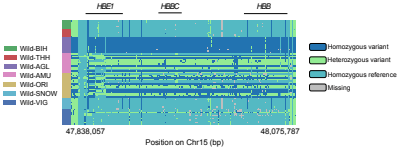

Supplement: qzae030_Supplementary_Data [file qzae030_supplementary_data.zip › Figure S7.pdf]

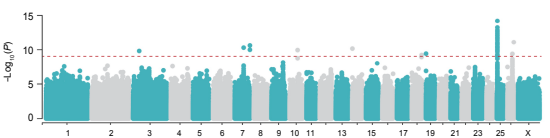

Supplement: qzae030_Supplementary_Data [file qzae030_supplementary_data.zip › Figure S8.pdf]

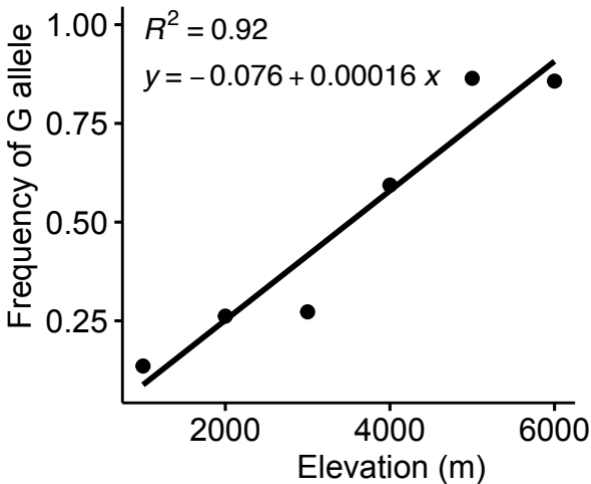

Supplement: qzae030_Supplementary_Data [file qzae030_supplementary_data.zip › Figure S9.pdf]

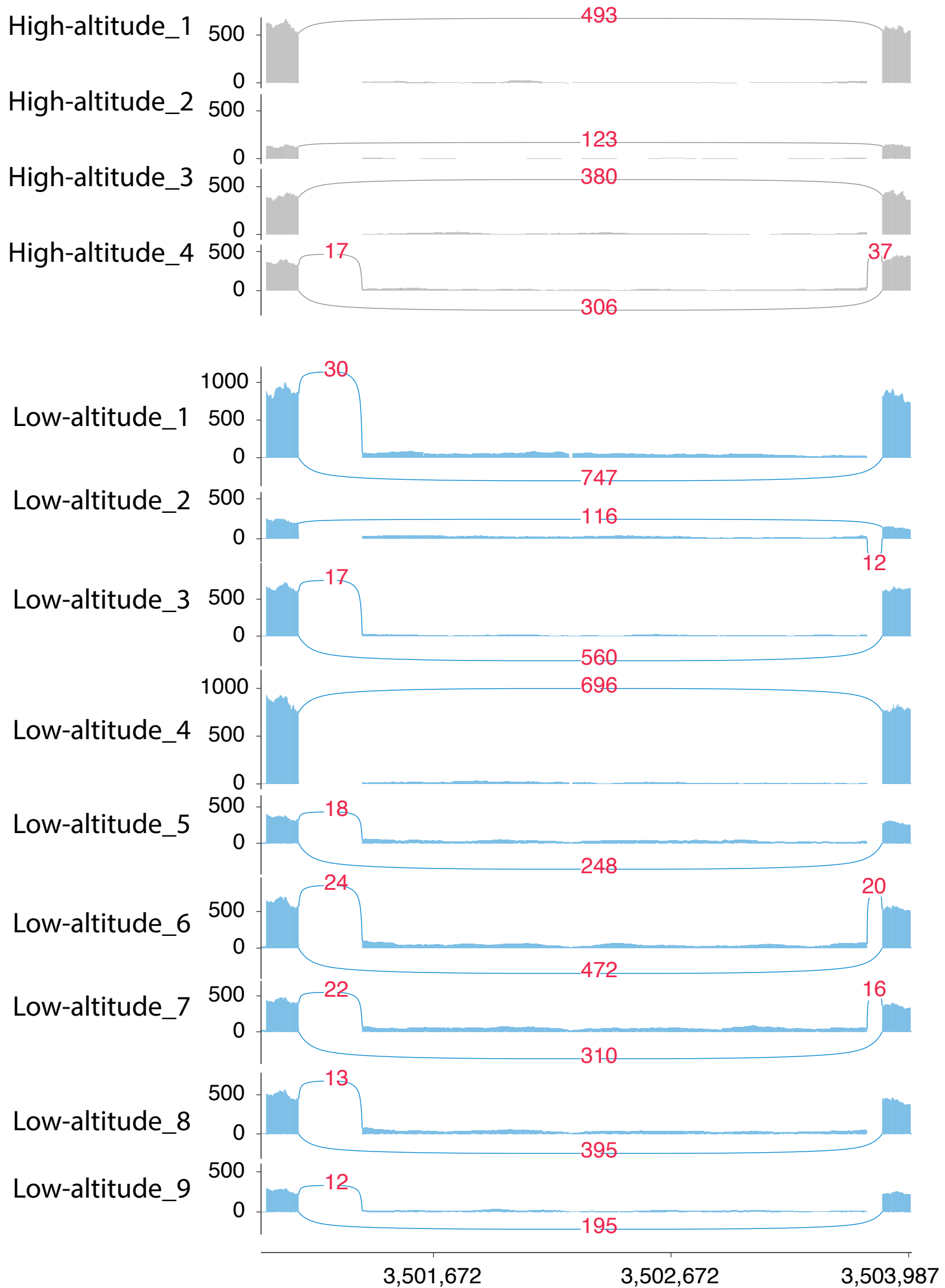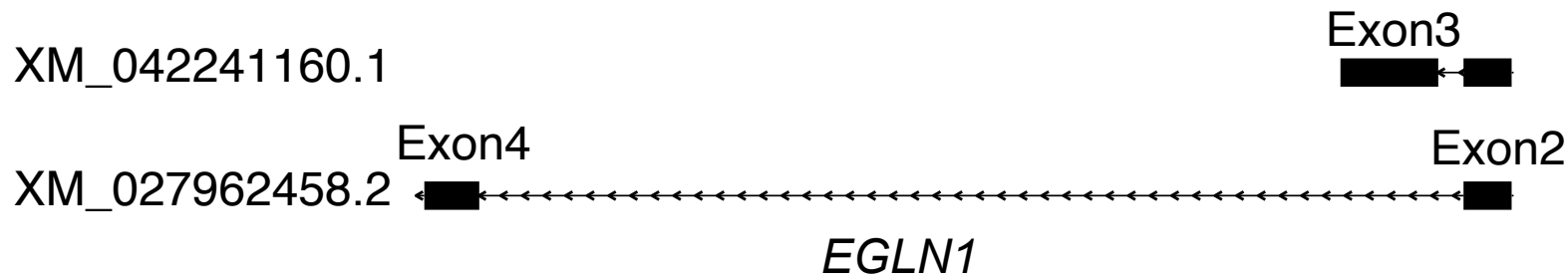

Supplement: qzae030_Supplementary_Data [file qzae030_supplementary_data.zip › Figure S10.pdf]

Exon2 Exon3

Exon4

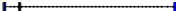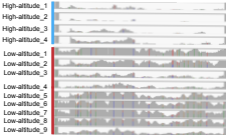

Supplement: qzae030_Supplementary_Data [file qzae030_supplementary_data.zip › Figure S11.pdf]

High-altitude

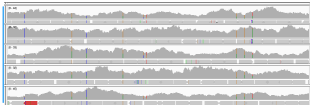

Low-altitude

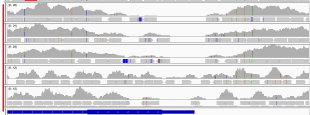

*EGLN1* exon1

Supplement: qzae030_Supplementary_Data [file qzae030_supplementary_data.zip › Figure S12.pdf]
